# Proteins in DNA methylation and their role in neural stem cell proliferation and differentiation

**DOI:** 10.1186/s13619-020-00070-4

**Published:** 2021-03-02

**Authors:** Jiaqi Sun, Junzheng Yang, Xiaoli Miao, Horace H. Loh, Duanqing Pei, Hui Zheng

**Affiliations:** 1 Bioland Laboratory (Guangzhou Regenerative Medicine and Health Guangdong Laboratory), #188 Kaiyuan Ave., Science City, Huangpu District, Guangzhou, 510700 China; 2 CAS Key Laboratory of Regenerative Biology, Guangzhou Institutes of Biomedicine and Health, Chinese Academy of Sciences, Guangzhou, 510530 China; 3 Guangdong Provincial Key Laboratory of Stem Cell and Regenerative Medicine, Guangzhou, 510530 China; 4 Institutes for Stem Cell and Regeneration, Chinese Academy of Sciences, Beijing, 100101 China; 5 School of Life Science, Westlake University, Hangzhou, 310024 China

**Keywords:** DNA methylation, Neural stem cells, DNA methyltransferases, Methyl-CpG binding proteins, Ten-eleven translocations, Vitamin C

## Abstract

**Background:**

Epigenetic modifications, namely non-coding RNAs, DNA methylation, and histone modifications such as methylation, phosphorylation, acetylation, ubiquitylation, and sumoylation play a significant role in brain development. DNA methyltransferases, methyl-CpG binding proteins, and ten-eleven translocation proteins facilitate the maintenance, interpretation, and removal of DNA methylation, respectively. Different forms of methylation, including 5-methylcytosine, 5-hydroxymethylcytosine, and other oxidized forms, have been detected by recently developed sequencing technologies. Emerging evidence suggests that the diversity of DNA methylation patterns in the brain plays a key role in fine-tuning and coordinating gene expression in the development, plasticity, and disorders of the mammalian central nervous system. Neural stem cells (NSCs), originating from the neuroepithelium, generate neurons and glial cells in the central nervous system and contribute to brain plasticity in the adult mammalian brain.

**Main body:**

Here, we summarized recent research in proteins responsible for the establishment, maintenance, interpretation, and removal of DNA methylation and those involved in the regulation of the proliferation and differentiation of NSCs. In addition, we discussed the interactions of chemicals with epigenetic pathways to regulate NSCs as well as the connections between proteins involved in DNA methylation and human diseases.

**Conclusion:**

Understanding the interplay between DNA methylation and NSCs in a broad biological context can facilitate the related studies and reduce potential misunderstanding.

## Background

Epigenetics, the study of heritable changes in gene functions without changes in the actual genetic or underlying DNA sequence mechanisms (Bird 2007), is a fundamental mechanism in modulating gene expression in a particular cell type during development. DNA methylation, one of the main epigenetic modifications, non-coding RNAs, and histone modifications are three predominant mechanisms. Particularly, in mammalian DNA, the principal modification is methylated cytosine, which is catalyzed by a family of DNA methyltransferase enzymes (DNMTs) (Goll and Bestor 2005) and predominantly occurs in CpG dinucleotide sequences to generate 5-methylcytosine (5-mC) on the pyrimidine ring (Zhu 2009). DNA methylation in terminally differentiated cells was believed to be irreversible until the discovery of ten-eleven translocation (TET) enzymes in 2009 (Pastor et al. 2013). TET enzymes, identified as key players in active DNA demethylation process, can convert 5-mC into 5-hydroxymethylcytosine (5-hmC) (Tahiliani et al. 2009; Ito et al. 2010) and subsequently into 5-formylcytosine (5-fC) and 5-carboxylcytosine (5-caC) (Ito et al. 2011). The functions of TET proteins in brain development attract much attention because the mammalian brain has the highest abundance of 5-hmC among all the tissues investigated thus far (Kriaucionis and Heintz 2009; Globisch et al. 2010).

In mammalian embryogenesis, central nervous system (CNS) development begins with the induction of neuroectoderm, which forms the neural plate and then invaginates to generate the neural tube. Neural stem cells (NSCs) arise from neuroepithelial cells and the radial glia lining the neural tubes and differentiate into three major cell types in the CNS in a temporally defined sequence, with neurons appearing first, followed by astrocytes, and oligodendrocytes (Temple 2001; Kriegstein and Alvarez-Buylla 2009). After the completion of the initial embryonic development, multipotent NSCs are largely restricted to two germinal zones in rodent model, namely the subgranular zone (SGZ) of the dentate gyrus in the hippocampus and the subventricular zone (SVZ) along the lateral ventricular wall (Kempermann and Gage 1999; Ming and Song 2011). In a mouse study, adult hippocampal neurogenesis, which shares embryonic dentate neural progenitors, was found to be a continuous process from development (Berg et al. 2019). NSCs in the adult CNS sparkle the field that these preserved cells can be harnessed to repair injured or diseased brain. Therefore, the regulation of the proliferation and differentiation of NSCs is an important area of research. Significant advances to identify the dynamic roles of DNA methylation in brain development have been made. In this review, we highlight DNA methylation and DNA demethylation, focusing on the modulation of the proliferation and differentiation of NSCs.

## DNA methylation in NSC proliferation and differentiation

### DNA methyltransferases

DNA methylation is a major epigenetic mechanism for gene inactivation; it serves as the basis of silenced X chromosome and the establishment of parental-specific imprints during gametogenesis (Jaenisch and Bird 2003; Reik 2007). DNA methylation is catalyzed by a family of DNA methyltransferases (DNMTs), including DNMT1, DNMT3A, and DNMT3B (Goll and Bestor 2005). DNMT3A and DNMT3B are de novo methyltransferases, which are responsible for the covalent addition of a methyl group from S-adenosyl-l-methionine (SAM) to the C5 of cytosine in CpG dinucleotides, whereas DNMT1 mainly recognizes the hemimethylated DNA and maintains DNA methylation patterns in dividing somatic cells (Law and Jacobsen 2010). However, the classification is not tightly restricted. Limited evidence suggests the presence of de novo activity of DNMT1 (Okano et al. 1998; Yarychkivska et al. 2018; Arand et al. 2012), but the overall evidence was not immediately clear. In 2018, Zhu’s group found the first systematic evidence confirming that DNMT1 functions as a de novo methyltransferase in vivo (Li et al. 2018). It has been reported that inhibiting DNMT1-mediated de novo methylation by Stella is important for establishing DNA methylation pattern during oogenesis (Li et al. 2018). Utilizing a “DNMT1-only” mouse embryonic stem cell line, a collaborative study by Xie’s and Zhu’s groups found that weak de novo methylation activity and imperfect maintenance methylation activity of DNMT1 can lead to spontaneous DNA methylation mutations (epimutations), which tend to be repaired by neighbor-guided correction based on the de novo and maintenance activities of DNMT1 in turn (Wang et al. 2020a). Additionally, the researchers found that the de novo and maintenance activities of DNMT1 vary in different genome regions, such as the enrichment of H3K9me2/3 areas with higher de novo activity and CpG islands with lower maintenance and de novo activities, thereby providing a new mechanism for maintaining different methylation levels in different regulatory sequences of the genome. Except for CpG methylation, other non-CpG sequences containing cytosine (CpA/C/T) can be methylated (hereafter referred to as mCpH) and have been found in embryonic stem cells (Lister et al. 2009; Ramsahoye et al. 2000) and neurons (Guo et al. 2014). Unlike most mCpG, mCpH is established during postnatal neuronal maturation in both the mouse and human brains. de novo methyltransferase DNMT3A has been shown to induce non-CpG methylation (Ramsahoye et al. 2000). A study of DNA methylation at single-base resolution in human has revealed that the highly conserved non-CpG methylation accumulates in neurons but not in glia during postnatal development, a critical period of synaptogenesis and neural circuit maturation (Lister et al. 2013). The growing knowledge on non-CpG methylation can shed new light on the function of DNA methylation in modulating brain development and plasticity.

Neurogenesis precedes astrogenesis in embryonic brain development. Therefore, inhibiting the expression of genes critical for astrocytes in NSCs is a key step in determining the neuronal differentiation of these cells. DNMT1 is vital in the switch from neurogenesis to gliogenesis during this process. DNMT1 deficiency in neural progenitor cells (NPCs) leads to DNA hypomethylation and precocious astroglial differentiation through enhanced regulation of the Janus kinase-signal transducer and activator of transcription (JAK-STAT) signaling (Fan et al. 2005). The conditional knockout of DNMT1 in mitotic CNS precursor cells via the Cre/loxP system resulted in extensive hypomethylation in daughter cells, and these daughter cells were rapidly depleted within 3 weeks of postnatal life. In contrast, the inactivation of DNMT1 in postmitotic neurons did not affect the levels of global DNA methylation and cell survival during postnatal life (Fan et al. 2001). Therefore, appropriate DNA methylation status is essential for both the maintenance and differentiation of NSCs during early development. A recent study in mouse embryonic fibroblasts has showed that DNMT1 expression positively correlates with the cell proliferation rate, largely depending on the length of G1 phase, to prevent passive DNA demethylation. When the function of DNMT1 was inhibited and the proliferation was accelerated, passive DNA demethylation on the promoters of pluripotency-related genes, including *Oct4*, *Nanog*, *Sox2*, *Esrrb*, *Cdh1*, and *Epcam*, accumulated, which resulted in the upregulation of pluripotency-related gene expression (He et al. 2017). The “stemness” or the self-renewal property of NSCs is associated with a cohort of pluripotency-promoting transcription factors, whereas their differentiation is related to neurogenic and gliogenic genes. As demonstrated in the study mentioned above, the inheritance of DNA methylation mediated by DNMT1 may participate in the regulation of the expression of genes relating to proliferation or differentiation, thus favoring or prohibiting certain cell fates of NSCs. This requires further studies.

Several studies have revealed a critical role for de novo DNA methyltransferase DNMT3A in regulating NSC proliferation and differentiation. DNMT3A knockout mice exhibit enhanced self-renewal of NSCs and impaired neuronal differentiation for postnatal neurogenesis (Wu et al. 2010). Mechanistically, DNMT3A-mediated proximal promoter DNA methylation and nonpromoter DNA methylation are essential for regulating proliferation and neurogenesis-related genes expression, respectively (Wu et al. 2010). Additionally, an in vitro study revealed that DNMT3A deficiency led to increased cell proliferation as well as precocious astrocyte and oligodendrocyte differentiation of NSCs (Wu et al. 2012). Behaviorally, mice with conditional knockout of DNMT3A in GABAergic inhibitory neurons displayed several symptoms including impaired learning and memory (Lavery et al. 2020), which is tightly associated with adult hippocampal neurogenesis (Ming and Song 2005). Global loss of mCpH induced by DNMT3A deficiency resulted in altered gene expression in striatum inhibitory neurons (Lavery et al. 2020). It is well known that GABA-releasing interneurons are an important component of the adult NSC niche (Bond et al. 2015). Whether DNMT3A deletion in inhibitory neurons regulates adult neurogenesis in a cell non-autonomous manner requires clarification.

### Methyl-CpG-binding proteins

DNA methylation exerts its biological function in at least two ways, i.e., by directly impeding the binding of transcriptional factors to gene promoter regions and by forming silenced states of chromatin via recruiting specialized transcription factors, namely methyl-CpG-binding proteins (MBPs) (Zou et al. 2012). There are three subgroups in the MBP family, including methyl-binding domain (MBD) proteins, zinc finger/Kaiso family proteins, and SET- and RING-associated (SRA) domain proteins (Jobe and Zhao 2017).

#### Methyl-binding domain family

The MBD family proteins include MBD1–5 and MeCP2 (methyl-CpG-binding protein 2) (Hsieh and Zhao 2016). Among these, MBD1 and MeCP2 have been comprehensively studied in the regulation of NSCs. Recent studies have showed that MBD1 is expressed in the NSCs of dentate gyrus in the adult hippocampus, and that MBD1 deficiency results in the accumulation of NSCs and the impairment of the neuronal lineage differentiation. Furthermore, transcriptome analysis showed that the MBD1 mutant causes the upregulation of genes related to cell differentiation, particularly those related to astrocyte lineage differentiation (Jobe et al. 2017). Additionally, MBD1 regulates NSC proliferation by directly binding to the promoter region of the basic fibroblast growth factor (bFGF), a potent mitogen for adult NSCs both in vitro and in vivo *(*Gokul et al. 2009*)*, resulting in the tight regulation of bFGF expression in neural stem/progenitor cells (NSPCs) (Li et al. 2008). These studies reveal the important function of MBD1 in stem cell maintenance, thereby providing novel insights into how DNA methylation preserves the multipotency of stem cells for subsequent differentiation. Thus, the intrinsic epigenetic mechanisms mediated by MBD1 play crucial roles in the modulation of proliferation and differentiation of NSCs.

MeCP2, whose mutation causes the neurological disorder Rett syndrome, is another major regulator of gene expression belonging to the MBD family. As MeCP2 shows increased expression with neuronal differentiation (Jung et al. 2003), MeCP2 controls neuronal maturation and dendritic arborization in both developing (Kishi and Macklis 2010) and adult brains (Smrt et al. 2007). One of its best known targets is the brain-derived neurotrophic factor (BDNF), a neurotrophic factor that facilitates the differentiation, maturation, and survival of neurons in the mammalian brain (Bathina and Das 2015). Studies have showed that MeCP2 suppresses *BDNF* gene expression by selectively binding to the BDNF promoter III. After neuronal activation and membrane depolarization, MeCP2 moves out of its binding site in the BDNF promoter, thus allowing transcription to proceed (Chen et al. 2003). Additionally, BDNF upregulation after neuronal activity involves the dissociation of the MeCP2-histone deacetylase-mSin3A repression complex from its promoter (Martinowich et al. 2003). Except for the regulation of neuronal maturation by MeCP2, a recent study has showed that MeCP2 plays a novel role in modulating the proliferation and differentiation of adult NSCs (aNSCs) in vivo and in vitro by epigenetically regulating the expression of miRNA, miR-137 (Szulwach et al. 2010). However, it is not clear whether MeCP2 could regulate other miRNAs. It is well established that MeCP2 interacts with mCpG through MBD to recruit a co-repressor complex (including histone deacetylases, transcriptional repressor mSin3A, and other chromatin-silencing factors) (Nan et al. 1998), linking with its transcriptional repression domain (TRD), thereby altering chromatin organization and preventing transcription (Jones et al. 1998; Skene et al. 2010; Nan et al. 1996). Except for the primary function of gene silencing, recent studies demonstrate that MeCP2 binds to 5-hmC, an oxidation product of 5-mC, to facilitate transcription in the brain (Mellen et al. 2012). In accordance with the unexpected role of MeCP2, several studies have revealed that MeCP2 is a gene activator (Yasui et al. 2007; Chahrour et al. 2008) with critical roles in transcriptional regulation in the mammalian brain. Most MeCP2-bound promoters are actively expressed in human SH-SY5Y neurons (Yasui et al. 2007). Additionally, gene expression studies performed in the mouse hypothalamus suggested that MeCP2 associated with the coactivator CREB1 (cyclic AMP-responsive element-binding protein 1) for transcriptional activation (Chahrour et al. 2008). In addition to binding mCpG and 5-hmC, recent data showed that MeCP2 can bind mCpH in neurons in vivo (Chen et al. 2015) and repress transcription in vitro (Guo et al. 2014). Furthermore, the mCpH binding sites for MeCP2 are mostly restricted to mCpApC and the repressive transcriptional regulation in the brain is tuned by MeCP2 binding at mCpG and mCpApC sites (Lagger et al. 2017). As a multifunctional epigenetic reader, it has become evident that MeCP2 is involved in higher order chromatin organization (Dhasarathy and Wade 2008). Chromatin compaction by MeCP2 and nucleosomal arrays can be abolished by R168X and R270X Rett syndrome (RTT)-causing MeCP2 mutations (Georgel et al. 2003). In addition, the MeCP2-mediated heterochromatin organization and chromatin loops are critical for transcriptional regulation in the brain, which becomes aberrant because of RTT-inducing MeCP2 mutations (Horike et al. 2005; Agarwal et al. 2011). Overall, several questions on how MeCP2 and other MBDs regulate the proliferation and fate choice of NSCs remain unanswered.

#### Zinc finger/Kaiso family

The zinc finger/Kaiso family includes three members, namely Kaiso/ZBTB33, ZBTB4, and ZBTB38, and have been shown to preferentially or specifically bind methylated DNA through three Krüppel-like C2H2 zinc fingers (Filion et al. 2006). Kaiso modulates transcription by binding to the methylated sequence mCpGmCpG. Moreover, it can also bind the nonmethylated sequence CTGCNA, the consensus Kaiso binding site. The ZBTB4 and ZBTB38 require only a single methylated CpG for binding. However, studies on the role of the Kaiso family in the regulation of brain development are limited. Kaiso-deficient mice were found to be viable and fertile, without any developmental defects or abnormal gene expression (Prokhortchouk et al. 2006). Both ZBTB4 and ZBTB38 exhibited high expression in the brain (Filion et al. 2006). Thus, further studies on whether and how these proteins regulate NSCs are needed.

#### SET- and RING-associated domain family

Ubiquitin-like containing PHD ring finger (UHRF) 1 and UHRF2 are two members of the SRA domain family. They prefer to bind hemimethylated DNA using their SRA domain and interact with other epigenetic factors, such as histone deacetylase 1 (HDAC1) and DNMT1 (Bronner et al. 2013; Unoki et al. 2004). UHRF1 (also known as Np95 or ICBP90) is critical for the maintenance of DNA methylation through cell division and is also involved in DNA damage repair. Recently, an intriguing study developed a new method for studying the dynamic DNA methylation maintenance process - Hammer-seq sequencing technology, and found that UHRF1-Ligase1 and PCNA-DNMT1 interactions contribute to rapid replication-coupled maintenance, whereas the UHRF1-H3K9me2/3 interaction and nucleosome occupancy specifically regulate replication-uncoupled maintenance during mitotic inheritance of methylation (Ming et al. 2020). However, these two methylation maintenance pathways are imperfect, leading to the gradual loss of methylation at some CpG sites during continuous mitosis, which may be an important mechanism for selective hypomethylation in aging and tumorigenesis (Ming et al. 2020). UHRF2, also known as Np97 or NIRF, is involved in cell cycle progression. A study investigating the expression pattern of UHRF1 in the mouse brain from developmental to adult stages has showed that UHRF1 is abundantly expressed in proliferative NSPCs during the developmental stage and in the hippocampus of adult brain (Murao et al. 2014). However, how UHRF1 functions in NSPC proliferation and differentiation warrants further research.

## DNA demethylation in NSC proliferation and differentiation

DNMTs are known as “writers” of DNA methylation, whereas MBPs are the “interpreters” of DNA methylation. What are the eraser proteins for DNA methylation? Cytosine demethylation can occur through a passive process, which takes place in the absence of functional DNMT1 activity during DNA replication (Ooi et al. 2009; Chen and Riggs 2011). A recent study has suggested that Gadd45 proteins couple with the DNA-excision-repair-based DNA demethylation mechanism to promote active DNA demethylation (Ma et al. 2009a). An activity-dependent immediate early gene *Gadd45b*, a member of the Gadd45 family, is required for the activity-induced DNA demethylation of specific gene promoters, which are crucial for adult neurogenesis, including BDNF and bFGF (Ma et al. 2009b). Correspondingly, *Gadd45b* knockout impaired the neural activity-induced proliferation of neural progenitors and the dendritic growth of newborn neurons in the adult hippocampus (Ma et al. 2009b).

In addition, DNA demethylation can occur actively via the enzymatic conversion of the methylated cytosine into cytosine without DNA replication. One well-known pathway of active demethylation is regulated by the TET family proteins, which catalyze 5-mC into an oxidized 5-hmC and subsequently into 5-fC and 5-caC (Tahiliani et al. 2009; Ito et al. 2010). TET family proteins have three members, namely TET1, TET2, and TET3, whose expression patterns differ in tissues. Interestingly, they are all expressed in the brain, with TET3 showing the highest level, followed by TET2 and TET1 (Szwagierczak et al. 2010). Correspondingly, the mammalian brain retains the highest concentration of 5-hmC, the oxidation derivatives of 5-mC by TET proteins. Therefore, TET enzymes play an important role in the regulation of NSCs in theory. A recent study has demonstrated that TET1 is highly expressed in NPCs in adult hippocampus, and that TET1-knockout mice show reduced progenitor proliferation, thus resulting in impaired spatial learning and memory (Zhang et al. 2013). In contrast, TET1 deletion has no effect in mouse development (Dawlaty et al. 2011) and, in NPCs, impacted a cohort of genes, which were hypermethylated and downregulated, involved in progenitor proliferation (Zhang et al. 2013).

During neurodevelopment, the expression dynamics of three TET dioxygenases are different. A significant and specific increase in TET2 expression, accompanied by a dramatic increase in global 5-hmC level, was found in differentiated aNSCs compared with proliferating aNSCs. In contrast, TET2 ablation stimulated aNSC proliferation and inhibited their neuronal and glial differentiation both in vivo and in vitro. Mechanistically, TET2 binds to the transcription factor Foxo3a and regulates the expression of genes related to aNSC proliferation and differentiation (Li et al. 2017). Thus, TET1 and TET2 possess distinct roles in the regulation of NSCs through different mechanisms. Another member of TET proteins TET3 is also critical in NPC maintenance and in the terminal differentiation of neurons (Li et al. 2015). An intriguing report has suggested a novel non-catalytic role for TET3 in regulating NSC maintenance within the adult SVZ (Montalban-Loro et al. 2019). Montalbán-Loro and colleagues discovered that TET3 directly binds to *Snrpn*, belonging to the Prader–Willi imprinted gene cluster, thus contributing to the transcriptional inactivation of the gene (Montalban-Loro et al. 2019). The interaction between TET DNA dioxygenases and other epigenetic regulators during neural development warrants further exploration.

## Mechanisms of opioids and vitamin C by TET1

**Fig. 1:**
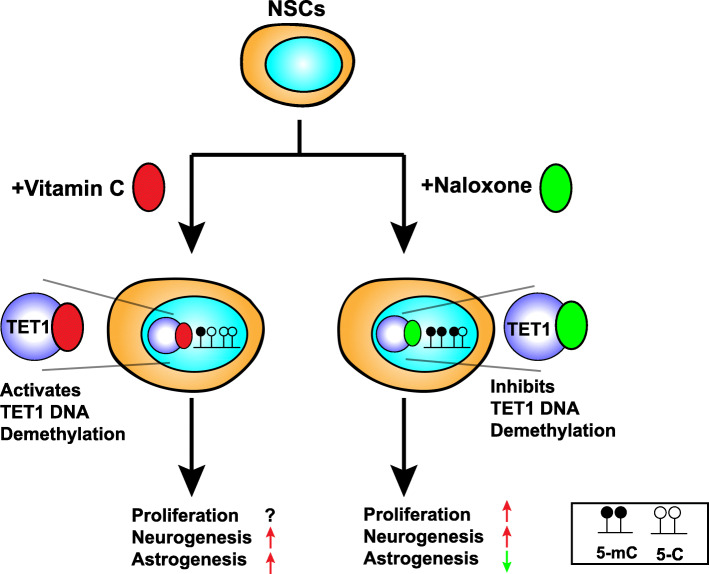
Small molecules modulate NSCs by regulating TET1 activity. Vitamin C (Vc) increases the DNA demethylation activity of TET1 and increase both neurogenesis and astrogenesis of NSCs. Naloxone decreases the DNA demethylation activity of TET1, increases proliferation and neurogenesis of NSCs, and decreases astrogenesis

Opioids, known as addictive drugs, have been studied extensively in NSCs (Eisch et al. 2000; Zheng et al. 2013). Our previous study showed that morphine blocks the CPP (conditioned place preference) training-induced enhanced survival of newborn neurons with no effect on early neural progenitors in adult hippocampus (Zhang et al. 2016). Because the expression of opioid receptors in NSCs is very low (Hutchins et al. 2017), the mechanism underlying NSC regulation by opioids is largely unknown. Our recent study has showed that the opioids morphine and naloxone, which can penetrate membranes, facilitate NSC proliferation via a receptor-independent and TET1-dependent pathway (Liang et al. 2020). These two opioids bound to the TET1 protein via three key residues (1880–1882) and suppressed the DNA demethylation ability of TET1, resulting in the reduced proliferation of NSCs. These results are consistent with that of a previous study on TET1 null mice. Additionally, the receptor-independent functions of naloxone and morphine were not observed in TET1-knockout NSCs. Furthermore, when the expression of opioid receptors was increased during the late stage of NSC differentiation, morphine but not naloxone, inhibited the neuronal differentiation of NSCs via a traditional receptor-dependent and miR181a-Prox1-Notch-related pathway (Fig. 1) (Hansen et al. 2011; Xu et al. 2014). These results demonstrate a direct link between opioids and epigenetic regulation in NSCs, thereby expanding our understanding on the regulatory mechanism of opioids onto NSCs.

In contrast to the impact of naloxone and morphine, some small chemicals, such as ascorbic acid (AA), enhance the catalytic activity of TET enzymes (Yin et al. 2013). AA (or vitamin C) can particularly bind to the catalytic domain of TET enzymes to enhance their oxidation capacity, leading to DNA demethylation. Thus, AA acts as a cofactor of TET enzymes (Yin et al. 2013). Emerging evidence demonstrates that AA-induced epigenetic regulation plays an important role in brain development. AA promoted the neuronal and astrocyte differentiation of CNS precursor cells in vitro *(*Lee et al. 2003*)*. He and colleagues recently discovered that AA enhances NSC differentiation into dopamine (DA) neurons. Mechanistically, AA induced the TET1-mediated DNA hydroxymethylation on the promoters of DA phenotype gene. Subsequently, Nurr1, a transcription factor critical for DA neuron development, was recruited to these gene promoters, thereby activating transcription (He et al. 2015). The same research group then showed that AA treatment induces the enrichment of 5-hmC near the consensus binding motifs of nuclear factor I (NFI) and enhanced the recruitment of NFI and STAT3 in the 5-hmC-enriched regions of astrocyte-specific genes, thereby leading to the upregulation of astrocytic genes and increased astrocyte differentiation (Kim et al. 2018) (Fig. 1). AA not only influences the catalytic activity of TET, but also plays a key role in determining the biological outcome of its function. In the presence of AA, TET1 negatively regulated somatic cell reprogramming by regulating 5-hmC formation at MET-related loci, whereas in its absence, TET1 promoted MET-independent somatic cell reprogramming (Chen et al. 2013). These studies deepen our understanding of the essential functions of epigenetic regulation in linking external chemicals to the nuclear transcriptional control of gene expression in NSCs and their progeny. However, the interaction of AA with epigenetic pathways to regulate NSC proliferation remains unknown.

## Connections between DNMT1 and TET1

**Fig. 2:**
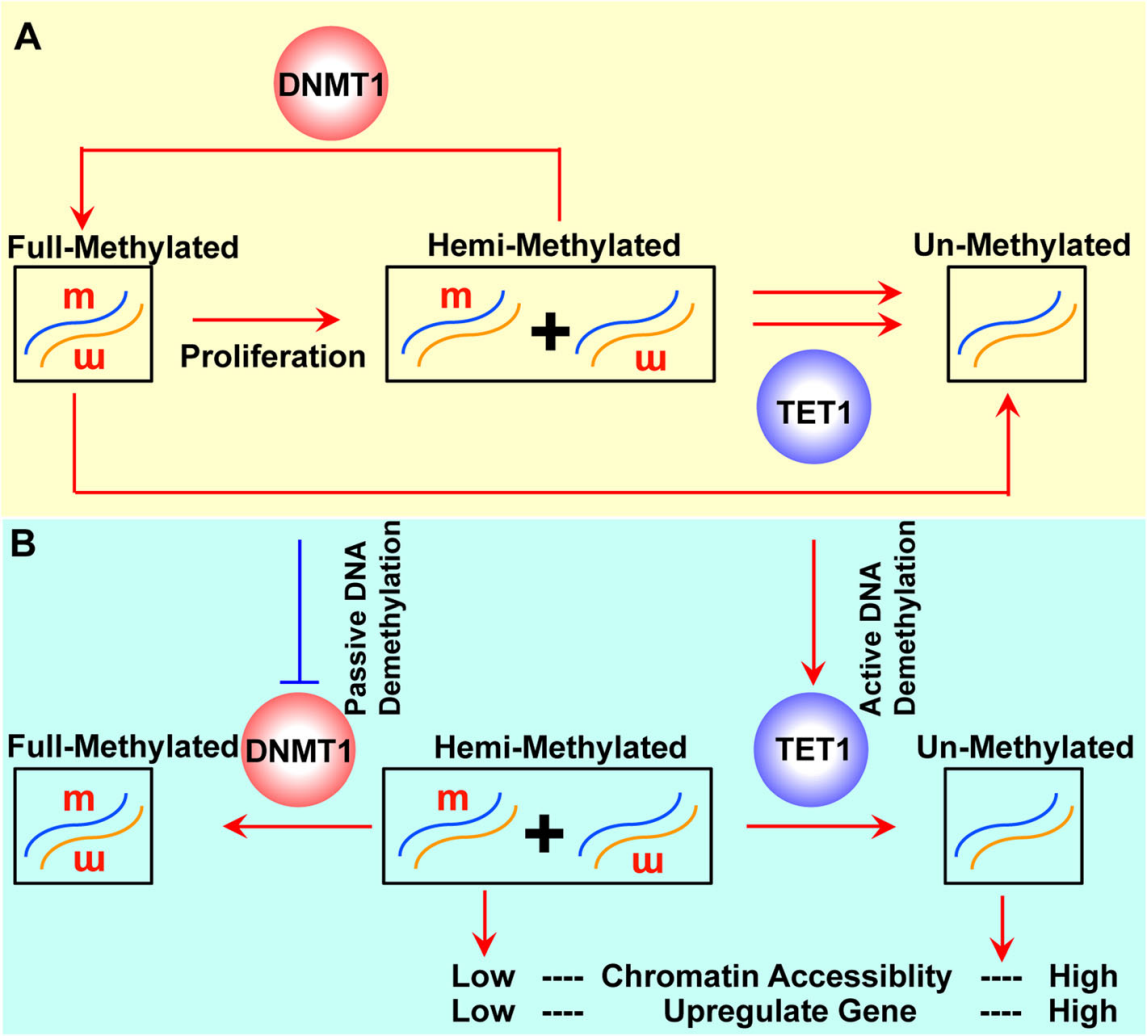
The connections between DNMT1 and TET1. **a** DNMT1 maintains DNA methylation during proliferation by methylating hemimethylated CpG sites into full-methylated. TET1 has higher abilities to demethylate hemimethylated CpG sites than full-methylated. **b** Passive DNA demethylation by inhibiting DNMT1 leads to lower increase in chromatin accessibility and gene expression than active DNA demethylation by stimulating TET1

We have thus far shown how DNA methylation and demethylation are closely linked. The connections between DNMT1 and TET1 have also been explored. DNMT1 preferentially recognizes hemimethylated DNA and remains activated in the G_1_ phase, following S and M phases, to ensure that DNA methylation is stably inherited across generations (Law and Jacobsen 2010). On the other hand, TET1 has showed greater ability to demethylate hemimethylated CpG sites than methylated CpG sites (He et al. 2019) (Fig. 2a). Therefore, DNMT1 and TET1 compete with each other for the hemimethylated CpG sites (He et al. 2019). Furthermore, the tight competition between DNMT3s and TETs at a large collection of methylated somatic enhancers is observed in both human and mouse pluripotent cells (Charlton et al. 2020).

Because the pluripotency-related and epithelial cell-specific genes are more likely to have unmethylated newly synthesized DNA strands after the S phase (He et al. 2017), the competition between DNMT1 and TET1 on these genes may play critical role in NSCs. This hypothesis is supported by three facts: 1) NSCs have high expression of several markers of pluripotency, such as *Sox2*; 2) NSCs proliferate at a relatively high rate; and 3) the differentiation from NSCs to neurons and astrocytes involves changes in the expression of mesenchymal and epithelial markers.

DNA demethylation includes passive DNA demethylation, which is normally induced by inhibiting the functions of DNMT1, and active DNA demethylation, which is normally induced by stimulating the functions of TETs. Although both kinds of DNA demethylation decrease methylation levels around the transcription start sites, passive DNA demethylation decreases fully-methylated CpG sites but increases hemimethylated CpG sites, whereas active DNA demethylation decreases hemimethylated CpG sites and increases unmethylated CpG sites. Because these two kinds of DNA demethylation have different abilities to affect chromatin accessibility and gene expression (He et al. 2019), they may play distinct roles even when induced in the same system (Fig. 2b).

Additional connections between DNMT1 and TET1 should also be discussed when AA is used to control TET1 activity or cell proliferation, when the epithelial–mesenchymal transition (EMT) or MET is induced, and when the switch between energy metabolic models (oxidative phosphorylation and glycolysis) is modulated (Sun et al. 2020).

## Abnormal DNA modifications and neurological diseases

Emerging evidences have showed that abnormal DNA methylation is associated with the pathophysiology of many neurological diseases.

Several studies suggest that aberrant DNA methylation is involved in many neurodegenerative diseases, including Parkinson’s disease (PD) and Alzheimer’s disease (AD) (De Jager et al. 2014; Coppieters et al. 2014; Chuang et al. 2017). By new base-resolution mapping and analytical technologies, a study has identified a group of AD-specific 5-mC, 5-hmC, and 5-fC/−caC signatures (Fetahu et al. 2019). In addition, DNA methylation and gene expression in schizophrenia, a severe psychiatric disease, have also been explored (Liu et al. 2014; Vitale et al. 2017). Five gene loci which are differentially methylated are common in different cell types deriving from schizophrenia patient. Collectively, these studies demonstrate that DNA methylation plays a pivotal role in neurological diseases and that the identified disease-specific epigenetic signatures can be implicated in the early diagnosis and prognosis of the diseases.

DNMT1 mutations lead to central and peripheral neurodegeneration. Thus, the patients present hereditary sensory and autonomic neuropathy with dementia and hearing loss, which arise from global hypomethylation and site-specific hypermethylation (Klein et al. 2011). In addition, mutations in DNMT3A have recently been associated with neurodevelopmental disorders. Through whole-genome sequencing, DNMT3A has been identified as a candidate gene marker for autism spectrum disorder (Jiang et al. 2013), and mutations in DNMT3A have been involved in overgrowth syndrome (Tatton-Brown et al. 2014; Tatton-Brown et al. 2018) and acute monocytic leukemia (Yan et al. 2011). In contrast, de novo missense mutations in the PWWP domain of DNMT3A result in microcephalic dwarfism due to failure of binding to H3K36me2 and H3K36me3, thereby altering DNA methylation of Polycomb-marked regions (Heyn et al. 2019). Thus, these findings provide a novel framework for the study of neurological disorders.

MeCP2 dysfunction, including loss of function and increased dosage, causes a range of neuropsychiatric disorders (Moretti and Zoghbi 2006). Numerous studies have shown that RTT, a neurodevelopmental disorder, is closely related to mutation of MeCP2 on the X chromosome (Moretti and Zoghbi 2006; Amir et al. 1999; Weaving et al. 2005). A large amount of data has revealed that reduced 5-mC binding by MeCP2 mutations in the MBD contributes to the pathophysiology of RTT (Agarwal et al. 2011; Kudo et al. 2003). Additionally, a recent study demonstrated that MeCP2 R133C mutation may cause the disease by inhibiting 5-hmC binding (Mellen et al. 2012). Moreover, in mouse models of RTT and MeCP2 duplication syndrome, transcriptional analysis revealed that MeCP2 null or duplication affected genes with higher MeCP2 binding and mCpH levels accumulating as neurons maturation, which may be the molecular mechanism for the delayed onset of Rett (Chen et al. 2015). Furthermore, RTT-inducing mutants failed to induce MeCP2-driven chromatin compaction (Nikitina et al. 2007). Until recently, a new view on how the mutation of this gene causes the occurrence and development of RTT has emerged. The latest discovery from Young et al. revealed that methylated DNA significantly promoted the phase separation of MeCP2, which can be disrupted by RTT-causing mutated MECP2 and lead to changes in heterochromatin formation and abnormal transcription of related genes, ultimately resulting in the occurrence of diseases (Li et al. 2020). Two other earlier studies (Wang et al. 2020b; Fan et al. 2020) reported similar findings, showing that MeCP2-mediated phase separation is critical for the pathology of RTT, which provides a new angle to drug development for the treatment of neurological diseases. Mounting evidences show that malfunctioning TET proteins and abnormal 5-hmC modifications are involved in the pathophysiology of different neurological diseases. The selective deletion of TET1 in nucleus accumbens neurons in adult mice produced antidepressant-like effects (Feng et al. 2017). Genome-wide mapping revealed that 5-hmC levels are inversely correlated with MeCP2 dosage, whose mutations cause Rett syndrome (Szulwach et al. 2011). In addition, abnormal 5-hmC levels participate in the etiology of fragile X-associated tremor/ataxia syndrome, a late-onset neurodegenerative disorder (Yao et al. 2014). Thus, further research on 5-hmC modifications and TET proteins in the context of neurodevelopmental disorders is urgently needed. These studies will advance the understanding of diseases and contribute to the development of effective therapeutic treatments for human neurodevelopmental disorders.

## Conclusions

Here, we reviewed recent studies on proteins involved in DNA methylation, focusing on the proliferation and differentiation of NSCs. We discussed the connection between DNMT1, which methylates hemimethylated CpG sites, and TET1, which demethylates cytosine, because these proteins are highly connected in the expression of pluripotency-related and EMT- or MET-related genes, as well as in the regulation of cell proliferation and energy metabolism, which have been demonstrated to play important roles in a variety of biological processes, including embryonic development, somatic cell reprogramming, and cancer progression. By using these two proteins and DNA methylation regulation as bridges, the newly explored mechanisms in these processes can be further the studies on the proliferation and differentiation of NSCs.

In addition, we have identified a TET1-dependent and receptor-independent pathway used by opioids, i.e., morphine and naloxone, to modulate the proliferation and differentiation of NSCs. The chronic or repetitive administration of opioids causes adverse side effects, including tolerance and addiction, which are highly associated with adult hippocampal neurogenesis and contextual learning (Eisch and Harburg 2006; Leuner et al. 2006). Opioid receptors and endogenous opioid peptides should be considered in future studies on DNA methylation and NSCs.

Increasing evidence suggests that DNMT3A and DNMT3B closely interact with H3K36 methylation through their PWWP domain to maintain the proper level of intergenic and intragenic DNA methylation, which plays critical roles in mammalian development and has been implicated in human developmental disorders and cancers. Two promising studies simultaneously revealed that DNMT3A was recruited to the H3K36me2-marked domain to regulate the establishment of intergenic DNA methylation (Xu et al. 2020; Weinberg et al. 2019). Aberrant levels of histone H3K36me2 lead to significant alterations in global 5-mC levels, which result from abnormal DNA hypermethylation by DNMT3A in the intergenic region (Xu et al. 2020). In contrast, missense-mutant (Y365C, I310N or W297del) DNMT3A in the PWWP domain, which causes Tatton–Brown–Rahman syndrome (TBRS), a childhood overgrowth disorder, abolishes its binding affinity towards H3K36me2 resulting in aberrant intergenic CpG methylation (Weinberg et al. 2019). Mice with DNMT3A carrying a D329A point mutation in the PWWP domain exhibit postnatal growth retardation. DNMT3A^D329A^ is selectively recruited to H3K27me3-marked chromatin, which leads to DNA hypermethylation in the H3K27me3-marked domain and de-repression of genes involved in hypothalamus development (Sendzikaite et al. 2019). Similarly, a strong interaction between DNMT3B with H3K36me3 is observed both in mouse (Neri et al. 2017; Lee et al. 2017) and human embryonic stem cells (Lee et al. 2017; Tan et al. 2019), which is important for regulating cell type-specific genes expression as well as cancer establishment and progression. However, the function of crosstalk between DNMT3s and H3K36 methylations in NSC regulation has not been determined.

Glioblastoma multiforme (GBM) is a common brain tumor in children and adults. However, paediatric GBM is biologically and mechanistically distinct from adult GBM (Jones et al. 2012; Sturm et al. 2012). It has been shown that two recurrent mutations (K27M and G34R/V) in *H3F3A* encoding histone H3 variant H3.3 are closely associated with numerous pediatric brain tumors (Schwartzentruber et al. 2012). Recently, global analysis of histone modifications revealed that K27M mutation led to significantly reduced amounts of H3K27me2 and H3K27me3 (Chan et al. 2013; Lewis et al. 2013). As discussed above, there is intense interaction of DNMT3s with histone modifications to regulate DNA methylation. It is promising to investigate whether altered histone methylations induced by H3.3 onco-mutations lead to tumorigenesis by reshaping DNA methylation.

This review focused on the role of DNA methylation, including the related proteins and small molecules, in the modulation of proliferation and differentiation of NSCs. As a common epigenetic modification, DNA methylation is highly linked to other epigenetic modifications, including non-coding RNAs and histone modifications, as well as to a variety of biological processes. Understanding the interplay between DNA methylation and NSCs in a broad biological context can facilitate the related studies and reduce potential misunderstanding.

## Abbreviations

AA: Ascorbic acid
BDNF: Brain-derived neurotrophic factor
CNS: Central nervous system
CPP: Conditioned place preference
DA: Dopamine
EMT: Epithelial–mesenchymal transition
HDAC1: Histone deacetylase 1
JAK-STAT: Janus kinase-signal transducer and activator of transcription
MBD: Methyl-binding domain
MBP: Methyl-CpG-binding proteins
MET: Mesenchymal–epithelial transition
NFI: Nuclear factor I
NIRF: Np95/ICBP90-like RING finger protein
NPCs: Neural progenitor cells
NSCs: Neural stem cells
SAM: S-adenosyl-l-methionine
SGZ: Subgranular zone
SRA: SET- and RING-associated
SVZ: Subventricular zone
TET: Ten-eleven translocation
